# Establishment and optimization of an *E. coli* urinary tract infection model in Göttingen minipigs with strain recovery and characterization

**DOI:** 10.3389/fimmu.2026.1842934

**Published:** 2026-05-18

**Authors:** Joyce Lübbers, Kaila Orlandini, Emily Podob, Jeffrey Fernandez, Jolaine M Wilson, Marjolein van Heerden, Jeroen Zeijpveld, Joan van Kregten, Peter T. Buckley, Germie van den Dobbelsteen, Kirsten L. Bair

**Affiliations:** 1 Bacterial Vaccine Discovery & Early Development, Johnson and Johnson, Leiden, Netherlands; 2 Infectious Diseases and Vaccines, Johnson and Johnson, Spring House, PA, United States; 3 Scientific & In Vivo Strategies, Johnson and Johnson, Spring House, PA, United States; 4 Preclinical Sciences and Translational Safety, Johnson and Johnson, Beerse, Belgium

**Keywords:** agglutination, bacterial load, minipig, serotyping, UPEC, urinary tract infection, UTI89, whole genome sequencing

## Abstract

**Introduction:**

Urinary tract infections caused by uropathogenic *E. coli* (UPEC) affect millions of individuals worldwide. Existing animal models used to study UTI treatment or vaccine efficacy have limitations and cannot accurately predict efficacy in humans. In this study we aimed to establish and optimize an intraurethral UPEC urinary tract infection (UTI) model in Göttingen minipigs with characterization of the strains recovered from the urine.

**Methods:**

Foley balloon catheters were used to challenge minipigs intraurethrally with UPEC strain UTI89. Urine collected after challenge was plated for bacterial enumeration and recovered bacteria was tested for agglutination and whole genome sequencing.

**Results:**

Through intraurethral challenge with UTI89, we were able to establish infection for 14 days. Agglutination and whole genome sequencing revealed that 75% of the animals were infected with UTI89 challenge strain, while in 25% of the animals, a different *E. coli* strain was recovered from urine. Therefore, our results emphasize the need for caution when interpreting bacterial enumeration outcomes from UTI models. Careful verification of strain identity is essential to ensure accurate evaluation of intervention effects with vaccine and antibiotic studies.

**Discussion:**

Altogether, we describe a novel minipig UTI model using an intraurethral challenge with UPEC strain UTI89 that can be used to assess vaccines and treatment efficacy; and will serve to bridge the gap between preclinical findings and clinical outcomes in humans.

## 1 Introduction

Urinary tract infections (UTIs) remain an unmet medical need affecting millions of patients annually while creating a huge economic burden (1–3). Antibiotic resistance is continuously increasing and impacts the ability of healthcare professionals to successfully treat UTIs (4). Left untreated, UTIs can progress into pyelonephritis and invasive *E. coli* disease (5, 6). Different animal models have been used to investigate UTIs. Murine UTI models have been used extensively to study the pathogenesis and mechanisms of infection of UTI (7, 8). However, they often fail to model the key anatomical, physiological, and immunological aspects of the human urinary tract (9, 10). Non-human primate (NHP) models have been developed to better translate preclinical findings to human UTI disease (9). However, ethical issues and the need for complex, costly environments make them difficult to use for all studies. This underlines the importance of finding alternative animal models to study UTIs.

Pigs and minipigs (*Sus scrofa domestica*) show promise as alternative models for studying human UTIs (11, 12). In terms of urogenital anatomy, renal physiology, and immunology, humans share more similarities with pigs and minipigs than traditional rodent models (13, 14). Comparable to humans, female minipigs have a shorter urethra when compared to their male counterparts (15). Moreover, female minipigs are naturally susceptible to uropathogenic *E. coli* (UPEC) strains, making them suitable for investigating UTIs (16). Natural infections in pigs are notoriously difficult to diagnose due to the lack of apparent clinical signs, however, diagnosis of UTIs is important as UTIs can reduce reproductivity in pigs. Diagnosis is performed by dipstick testing or urine culture if clinical signs are apparent, however, most UTIs go undiagnosed and are only detected post mortem (17, 18). Göttingen minipigs were chosen for this study instead of farm pigs because of their defined genetics, known health history, and smaller sizes, which is more amenable to a laboratory setting. Most experimental murine and pig UPEC UTI studies employ trans urethral inoculation by passing a catheter into the bladder and depositing the bacteria directly into the bladder (8, 12). In our studies, the urethra was inoculated with bacteria, more closely mimicking the natural route of infection as it allows the bacteria to migrate to the bladder. UPEC strain UTI89, a human cystitis strain, was used as it causes cystitis in both humans and pigs.

In any animal infection model, it is essential to identify and characterize the bacterial strain responsible for disease to ensure it corresponds to the original challenge strain. This requirement is particularly critical in models such as the pig and minipig, where naturally occurring infections by the same pathogen species as those under investigation may confound results. Additionally, over the course of UTI animal model experiments, polymicrobial colonization and environmental contamination is possible, therefore, verifying the identity of the challenge strain in recovered urine and tissues is especially important. Challenge strain verification has been achieved through various methods, including the use of fluorescently labeled bacteria, which can be subsequently detected and visualized within bladder wall biopsies (12).

In our studies, we employed whole-genome sequencing, O-antigen serotyping, and assessment of phenotypic traits such as hemolysis on a blood agar plate to identify whether the recovered organism was the intended pathogen. As minipigs have been widely utilized in multiple disease areas, including infectious diseases such as tuberculosis and influenza, as well as metabolic and cardiovascular disorders like diabetes and atherosclerosis, they could present a relevant and appropriate translational model for the study of UTI (12, 19).

This paper explores the utility of the Göttingen minipig as a translational model for human UTIs and highlights methods for confirming strain identification of bacterial isolates recovered from urine. Rigorous strain characterization, combined with the intrinsic anatomical and immunological similarities between pigs and humans, strengthens the translational relevance of this model and will serve to bridge the gap between preclinical findings and clinical outcomes in humans.

## 2 Materials and methods

### 2.1 Animals and housing

Five independent studies were performed with sexually mature, female Göttingen minipigs (Marshall BioResources, USA) ranging from 5 to 9 months of age. Upon arrival, minipigs were housed in accordance with the *Guide for the Care and Use of Animals* and Animal Welfare Act and in an AAALAC-accredited facility under controlled temperature and humidity and maintained on a 12-hour light/dark cycle with ad libitum water. All procedures were conducted under an Institutional Animal Care and Use Committee (IACUC) approved protocol by Janssen Research & Development Spring House, Pennsylvania, USA. Minipigs were acclimated for a minimum of 2 weeks. During this time, minipigs received positive reinforcement to familiarize them with handlers and equipment that they would encounter during the study. This process is intended to reduce stress experienced by the animal at the time of the study to achieve more reliable and accurate results. Minipigs were group-housed and separated as needed for fasting, urine collection, or aggression. In addition to social enrichment, the minipigs were provided with dietary, occupational, and cognitive enrichment.

Body weights were captured upon arrival and as needed prior to sedation and for the duration of infection to monitor animal health. Peripheral body temperatures were also monitored for animal health prior to challenge and for the duration of infection via implanted BMDS transponders (Avidity Science).

### 2.2 Bacteria

UTI89 is a human cystitis-derived UPEC isolate of serotype O18:K1:H7, commonly used in other *in vivo* UTI models (12, 20), and kindly provided by T.E. Andersen. Multiple glycerol stocks were created from this parent isolate, with one stock used per study then discarded. One 10µL loopful of UTI89 from this frozen glycerol stock was used to inoculate 50mL of LB in a vented flask, grown overnight at 37 °C and 200 RPM. The culture was centrifuged (Thermo Fisher, Megafuge 40R) for 10 minutes at 5000 RPM (± 4500 RCF). The supernatant was discarded, and the remaining culture was washed with sterile saline and the pellet resuspended in sterile saline. This suspension was adjusted to an optical density (OD) of 0.5, equaling approximately 1x10^8^ bacteria per minipig, and further serially diluted to achieve target inoculum concentration considering a 100µL inoculum volume. When targeting higher inoculum concentrations, an optical density of 1.0, approximately 1x10^9^ bacteria per minipig, was used. Quantification of bacterial inoculum (see section 2.5) was performed for each inoculum used in the minipig experiments.

### 2.3 Intraurethral challenge with balloon occlusion

Animals were sedated via intramuscular injection with 8 mg/kg Ketamine (Dechra) + 0.08 mg/kg Dexmedetomidine (Dexdomitor^®^, Zoetis) and maintained on (1-4%) isoflurane inhalant anesthesia and oxygen throughout the procedure. Heat support was provided, and physiological parameters were monitored for the duration of the procedure. The animal was placed in dorsal recumbency. Sterile preparation of the urogenital area was performed by removing hair and prepping the area with chlorhexidine. The area was sterilely draped, and a speculum was inserted through the vulva to visualize the urethral opening. A urinary catheter was passed through the urethra into the bladder to empty the bladder of urine and was then removed. Urine samples were sterilely handled and stored on ice until processing. A Foley catheter (Jorgen Kruuse) that was primed with inoculum, was inserted approximately 3 cm into the urethra opening, the balloon was inflated to prevent backflow, and 100µL of bacterial inoculum or sterile saline (Mock study) was injected. The animals remained under isoflurane inhalant anesthesia with the ballooned catheter in place. The animal remained in dorsal recumbency with the hind inclined slightly to prevent leakage of the inoculum. After 30 minutes, the balloon was deflated and the catheter was removed. Animals were given 0.8 mg/kg atipamezole (Antisedan^®^, Zoetis) via intramuscular injection to reverse Dexmedetomidine and were returned to their cage under continuous observation for full recovery.

### 2.4 Sample collection and infection monitoring

Urine collections were performed at various timepoints, just before and following intraurethral inoculation. Animals were sedated via intramuscular injection with 3–4 mg/kg of tiletamine and zolazepam (Telazol^®^, Zoetis). The same procedures for anesthesia, sterile preparation, and placement of the catheter were followed as described for intraurethral inoculation. Approximately 10mL of urine was removed, sterilely handled, and stored on ice until processing. The animals were returned to their cage under continuous observation for full recovery.

Prior to terminal urine and tissue collection (day 7, 14 or 28 post-challenge), animals were sedated via intramuscular injection with 0.044 mL/kg of a mixture of 50 mg/mL each of tiletamine, zolazepam, ketamine, and xylazine (Dechra). They were then prepared for sterile urine collection as previously described. After urine samples were collected, the animals were humanely euthanized, and tissues from urinary and genital tracts were evaluated and collected. Tissues were sampled for bacterial quantification and histology.

### 2.5 Bacterial quantification

Bacterial quantification of the inoculum, urine, and tissues (bladder, urethra, ureters, vagina, kidneys) was completed immediately following inoculum preparation or sample collection. Tissue sections were homogenized in 10mL of saline using a Bead Ruptor Elite (Omni International). A neat sample was plated by manually spreading 100µL on MacConkey Agar plates (Northeast Lab Services). Serial dilutions were performed and plated on MacConkey Agar using a WASP spiral plater (Don Whitley Scientific Limited). The plated samples were incubated overnight at 35 °C, and the colonies were counted using a Protocol3 plate reader (Microbiology International).

### 2.6 Histopathology

Histopathology was performed on urogenital tissues collected. The kidneys, ureters, urethra, urinary bladder (ventral and dorsal sections from the caudal region), and the vagina were sampled and fixed in 10% formalin. Tissues were processed at Johnson & Johnson PSTS, Spring House with a Vip 6- Robin tissue processor (Sakura TissueTEK) with a large animal procedure that included 8–72 hour incubation with 10% neutral buffered formalin solution (VWR, #89370-094), dehydration steps with ethanol (70% up to 100%, VWR) for a total of 7 hours, 2 hours in Xylene (Epredia, #9900-1) and 2 hours in Paraffin (Surgipath, #39601006). Sections were cut at 5-micron thickness with a microtome (Microm). Sections were heated in an oven for 10–30 min at 60 °C to remove paraffin. Sections were then stained with hematoxylin and Eosin staining using the Tissue-Tek Prism Plus Automatic Slide Stainer (Sakura TissueTEK). Slides were removed from the stainer, a coverslip was applied, and slides were scanned using a GT450 scanner (Leica), uploaded, and evaluated in HALOLink. Microscopic findings were either graded as 1: minimal histological change, 2: mild, 3: moderate, 4: marked, or 5: severe. Tissues were graded by a pathologist (not blinded) and scoring was confirmed by a secondary staff member. The Ascentos system was used to enter histopathology data and for reporting.

### 2.7 ELISA

O18 specific IgG serum antibody titers were evaluated by ELISA on serum samples isolated on day 0, 7, and 14 post challenge in 384-well plate format, where plates were coated with 30 µL per well of 2.5 µg/mL purified LPS obtained by single-phase extraction from representative O18 Extraintestinal Pathogenic *Escherichia coli* (ExPEC) isolates (LPS Biosciences) and 5 µg/mL methylated bovine serum in PBS. Plates were blocked with diluted skimmed milk in PBS with 0.05% Tween 20. Serum samples were added in a 12-step 3-fold serial dilution starting from 1:90. HRP-labeled goat-anti-pig secondary antibody (Sigma Aldrich #A5670-1mL) was used at 1:1000 dilution. Plates were developed using TMB substrate (Leinco). After adding 1M H_3_PO_4_ stop solution, plates were read at 450nm in a plate reader. Analysis was performed using Gen5 software v3.04 (Agilent). Optical density at 450nm was analyzed in a 4 parameter (4PL) nonlinear regression model. Half maximal effective concentration (EC50) was calculated for each individual sample based on duplicate 12-step titration curves. Sample results were expressed as EC50 titers. Values at or below the lower limit of detection (LLOD) were set to the LLOD of 30 EC50.

### 2.8 Bacterial glycerol stock generation for whole genome sequencing and agglutination

Two individual bacterial colonies were isolated from MacConkey plates (Northeast Lab Services) used for CFU enumeration of urine samples and each inoculated into 5mL of Lysogeny broth (Teknova). Cultures were incubated overnight shaking (200 RPM) at 37 °C. After incubation, 80% glycerol (MP Biomedicals, final concentration 20%) was added per tube, mixed, aliquoted into cryovials, and stored at -80 °C.

### 2.9 Whole genome sequencing

Bacterial sequencing results were derived from scraped MacConkey plates or glycerol stocks of isolates recovered from urine. All the isolates, were replated on blood agar plates (Hardy Diagnostics) and incubated overnight at 37 °C. Scrapes (from MacConkey agar plates) and isolates (replated on blood agar plates) were processed through a genomic DNA isolation protocol which included bead beating and solid-phase reversible-immobilization (SPRI) based DNA cleanup. A half-loopful (10μl inoculation loop) of colonies was transferred to bead tubes (Invitrogen A29158) containing 400μl nuclease fee water. Bead tubes were incubated for 20 minutes on a Vortex Genie 2 (Scientific Industries with vortex adapter, speed 6) and centrifuged for 2 minutes at 20,000g. Supernatants were transferred without the glass beads to a 96 well plate. 20μl of supernatant was combined with 30μl Nuclease free water and 20μl Illumina purification Beads, mixed, incubated for 5 minutes at RT, washed with 80% ethanol, airdried and eluted with 32.5μl Illumina Resuspension Buffer for 2 minutes. After the DNA cleanup, the Illumina Library Prep procedure was performed according to the manufacturer’s instructions. Illumina tagmentation was performed on approximately 100ng of input DNA. Tagmented DNA was amplified using a limited-cycle PCR protocol consisting of 20μl Illumina Enhanced PCR Mix, 20μl nuclease free water and 10μl tagmented DNA, incubated included: 3 minutes at 68 °C, 3 minutes 98 °C, 5 cycles (98 °C for 45 seconds, 62 °C for 30 seconds, 68 °C for 2 minutes), followed by 1 minute at 68 °C. The Index 1 (i7), Index 2 (i5) adapters and sequences required for sequencing cluster generation were added. Dual indexed Libraries using Illumina DNA/RNA UD indexes (20091646 ILMN Set A; a unique index was used per sample in each sequencing run) were used. Libraries were quantified using a Qubit 4 fluorometer and size distribution was measured using the Agilent TapeStation. Quantification results for individual samples were used for library molarity calculation to ensure an equimolar ratio during pooling. After pooling, library quantity was examined by a Qubit 4 fluorometer to confirm library concentration. PhiX control material was spiked in at 1%. Batch sizes for each sequencing run were within ranges validated to achieve a minimum of 50-fold average coverage of assembled *E. coli* genomes.

MiSeq v3 reagents kits were used to sequence samples on an Illumina MiSeq. The read type was set to paired-end with a read length of 2x300 (600 cycle). Raw sequencing reads were sorted into different sample files according to their unique molecular indexes and generated the FASTQ data files. Sequencing QC was performed with Sequencing Analysis Viewer (SAV) v1.11.1 Software. A run was accepted if an average of ≥70% of bases having a quality score of at least 30 was achieved.

CLC Genomics workbench version 25.0 (Qiagen) was used for read trimming and *de novo* genome assembly. Paired-end reads were trimmed by quality and read length, and sequencing adapters were removed. *De novo* assembly was performed from trimmed reads to construct contigs and scaffolds, using *de novo* assembly tool of the Qiagen CLC Genomic Workbench with word size (k-mer) 20, bubble size 50, minimum contig length of 500bp, scaffolding and auto-detection of paired distances. Following assembly, assay acceptance criteria for *E. coli* isolates were applied, including GC% within a range of 50.0%-51.2%, assembly size within a range of 4.3-6.0Mb, and mean genomic coverage of ≥50-fold. Mean assembly coverage was determined by mapping the reads back against the *de novo* assembled genome.

O-genotyper (version 4; a validated computational pipeline proprietary to Johnson & Johnson) was used to identify predicted O-serotypes from *E. coli* WGS assemblies with a blast-based approach against a reference database of the O-serotype-specific *wzy*, *wzx*, *wzt*, and *wzm* gene sequences utilizing the Abricate tool (https://github.com/tseemann/abricate). This reference database contains *wzy*, *wzx*, *wzm*, and *wzt* genes from 188 different *E. coli* O-serotypes. Sequences included in the reference database are based on the genes from Iguchi et al. (21), the SSI database, and genes from DebRoy et al. (22), with additional genes from newly identified O-serotypes. The Abricate tool was used to find *wzy*, *wzx*, *wzt*, and *wzm* genes from the O-genotyper reference database in the *de novo* assembled *E. coli* genomes and then parsed the output to assign an O-serotype. Predicted O-serotypes were assigned following the positive identification of at least one *wzy* or, *wzx*, *wzt*, and *wzm* gene specific for the O-serotype, with a threshold for sequence identity set at 80% or higher. O2 and O50 are 100% identical in identity for the *wzx* and *wzy* genes, requiring an additional step in the O-genotyper to differentiate these O-serotypes. To this aim, two sequences from Delannoy et al. (23) were used by the Abricate tool. Detection of two or more *wzy*, *wzx*, *wzt*, and *wzm* genes belonging to different O-serotypes, sequence identities lower than 80%, or detection of genes specific to *Shigella spp*, generates the output: ‘Not Determined’.

### 2.10 Agglutination

High throughput clinical validated agglutination assay for the ExPEC O serotypes O1, O2, O4, O6, O15, O16, O18, O25, and O75 was performed on isolates (glycerol stocks) cultured from day 0 and day 7 post-challenge minipig urine. One cryovial per animal was streaked on a 6-well veal infusion yeast agar plate (20g veal HiMedia, 1g yeast ThermoFisher, 0.5% dextrose Millipore Sigma in 500mL dH20) and incubated at 35 °C for 16–20 hours. A bacterial suspension was prepared in formal saline solution (0.216% formaldehyde Millipore Sigma, 5.5g NaCl Millipore Sigma, dH2O Corning) by adding bacterial colonies from the agar plates (10µL loop) into 96-well glass tubes (Chrom Tech) reaching an OD_600_ between 0.6-1.2. The bacterial suspensions were heat inactivated for 2 hours at 95 °C (to ensure proper heat inactivation), cooled to room temperature, the 50µL suspension added to antisera or control plates, and then incubated overnight at 50-52 °C, 0% CO_2_. Antisera plates were filled with 50µL of O1 (#81356), O2 (#81359), O4 (#81362), O6 (9#81357), O15 (#81361), O16 (#81379), O18ac (#81365), O25 (#81369) or O75 (#81478) antisera (all from Statens Serum Institute Diagnostics) and diluted according to manufacturer’s instructions with 0.85% saline solution (Millipore Sigma). Control plates were filled with 50µL 1x PBS (Growcells). Technical replicates of the samples were performed for each experiment. Plates were read with CTL Elispot reader (ImmunoSpot by C.T.L.) and total intensity of spots per well (TIOSPW) was determined. TIOSPW below 11.65 were marked as negative and above this point as positive. Specific positive control strains for each serotype were taken along as positive controls for the assay.

### 2.11 Statistics

GraphPad Prism 10.1.2 was used for statistical analysis. Wilcoxon matched pairs signed rank test for non-parametric paired samples was performed on ELISA EC50 data comparing day 14 with day 0. P values <0.05 were considered to be significant.

## 3 Results

### 3.1 Intraurethral inoculation of minipigs to mimic natural bladder infections

When developing a UTI model intended to investigate novel UTI vaccines or test new therapeutics or antibiotics, it is important that the model shows similarities in urogenital anatomy and renal physiology when compared to humans and mimics a natural infection route. To this end, we developed a minipig UTI model where the inoculum is delivered to the urethra of the animal instead of delivering bacteria directly into the bladder, as used in developed UTI models using farm pigs, mice, or rats (10, 12, 24). By delivering into the urethra, we stimulated the natural flagellum mediated mobility of *E. coli* (25), whereby the bacteria adhere to and ascend the urethra towards the bladder, a process that is bypassed by direct bacterial delivery into the bladder.

**Figure 1:**
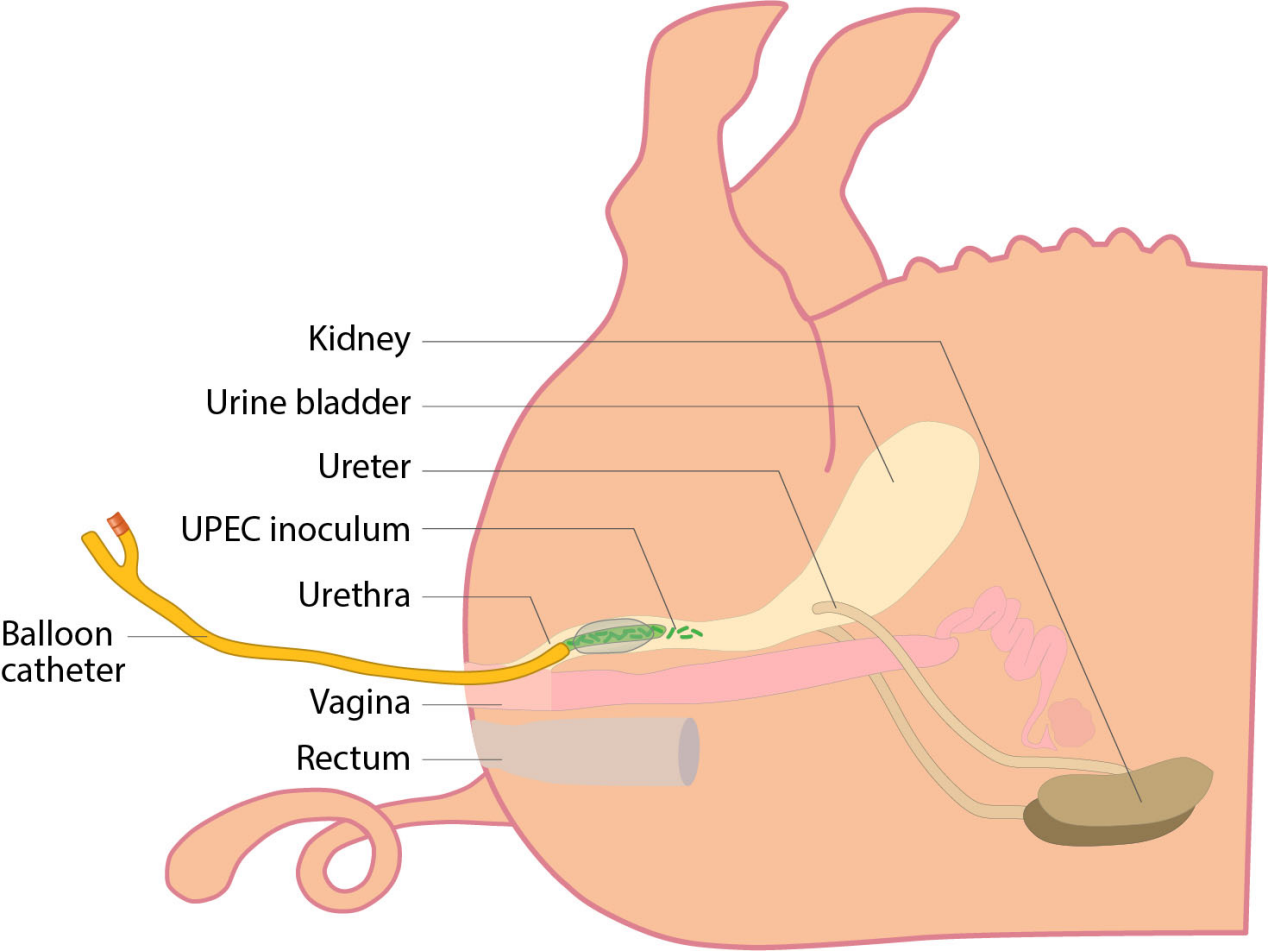
Schematic representation of the UTI minipig model with intraurethral inoculation with a Folley urine catheter and balloon occlusion. The catheter was placed approximately halfway into the urethra, the balloon inflated and the UPEC inoculum delivered.

In our UTI model using Göttingen minipigs, a foley catheter with an adapter containing the UPEC strain UTI89 was placed approximately 3 cm into the urethra, halfway of the total length of the urethra, and the foley balloon inflated to prevent leakage of the inoculum after it was delivered (**Figure 1**). To simulate a clinical infection, only a small inoculum volume of 100 µL was used. Therefore, our minipig UTI model was more closely able to mimic a natural route of infection with the UPEC strain UTI89.

**Figure 2:**
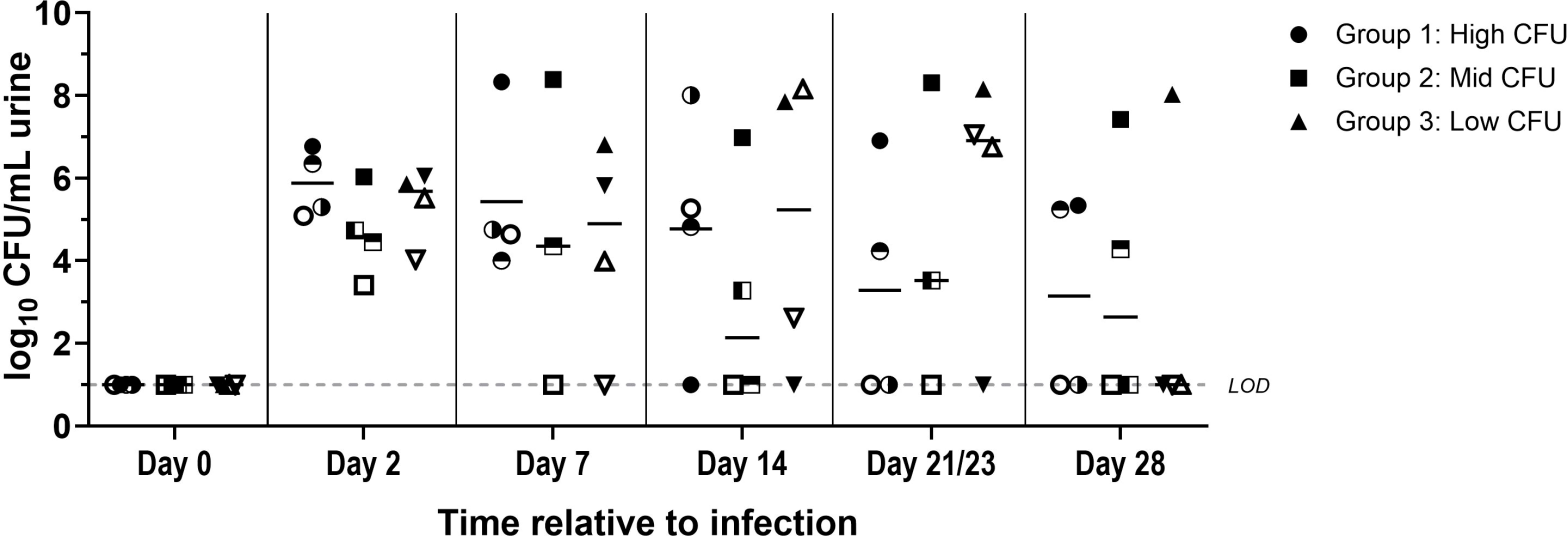
Bacterial quantification of urine in minipigs challenged with different amounts of UPEC strain UTI89 (study 1). The high CFU group (circles, n=4) received approximately 9 log_10_ CFU (range 8.80-9.05 log_10_ CFU), the mid CFU group (squares, n=4) received approximately 8 log_10_ CFU (range 7.80-8.05 log_10_ CFU), and the low CFU group (triangles, n=4) received approximately 7 log_10_ CFU (range 6.80-7.05 log_10_ CFU). Individual animals within a group can be tracked with the different filling of the symbols. Bacterial burden in the urine was quantified prior to challenge on day 0 and on days 2, 7, 14, 21, and 28 post-challenge. LOD, Limit of detection.

To determine the appropriate inoculum concentration to induce consistent infection in this UTI minipig model, we performed an inoculation titration experiment, Study 1 (**Supplementary Table 1**). Minipigs (n=4 per group) were challenged intraurethrally with approximately 9 log_10_ CFU (Group 1), 8 log_10_ CFU (Group 2), or 7 log_10_ CFU (Group 3) of UPEC strain UTI89. Measurement of the urine CFU indicated infection in all animals, with 100% of the minipigs presenting with quantifiable bacterial load on day 2. In the urine, natural clearance over time still resulted in a 42% infection rate on day 28 across the various groups, with at least one animal from each group sustaining infection (**Figure 2**). No differences in infection rate were observed between the groups.

Bacterial burden in the bladder, urethra, left ureter, right ureter, left kidney, right kidney, and vagina was quantified on day 28 post-challenge. Bacterial quantification of these tissues indicated no dose-dependent response, with a majority of animals failing to produce a quantifiable bacterial load in most tissues (**Supplementary Figure 1**). However, histopathology findings suggestive of a lower urinary tract infection were present in the urinary bladder, urethra, and kidneys of all groups with a distinct dose-dependent response between them. In the urinary bladder, an increased severity and incidence of histopathology findings like mucinous metaplasia of urothelium, inflammation, and edema were observed in Group 1 while findings in Groups 2 and 3 were relatively comparable (**Supplementary Table 2**). In the kidneys, a higher incidence of UTI89 infection-related histopathology findings like pyelonephritis and glomerulonephritis were observed in Groups 1 and 2, when compared to Group 3. Additionally, the urethra showed high inflammation in most of the animals of Group 1 (n=4/4) and 2 (n=3/4) compared to only one animal (n=1/4) in Group 3 (**Supplementary Table 2**). Overall, it was found that a 7 or 8 log_10_ CFU inoculum concentration would be able to establish an infection without causing major tissue damage as observed in the high inoculum group (9 log_10_ CFU).

### 3.2 Duration of infection influences bacterial load in urine and damage in bladder tissue

**Figure 3:**
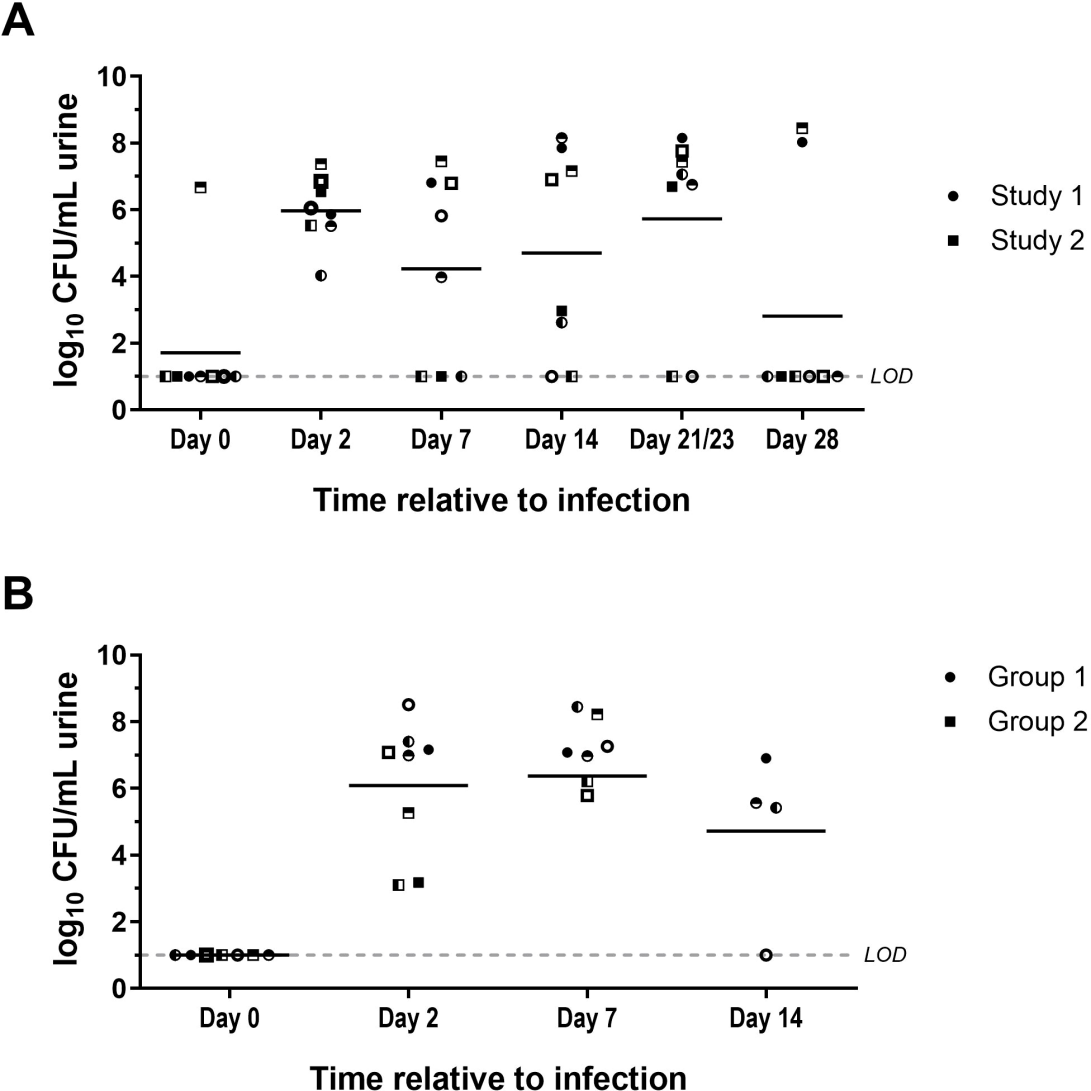
Bacterial quantification in urine of minipigs challenged with approximately 7 log_10_ CFU UTI89. **(A)** Bacterial burden in the urine was quantified prior to challenge on day 0 and on days 2, 7, 14, 21 (Study 1)/23 (Study 2), and 28 post-challenge in two different studies. Study 1 (n=4) depicted with circles and Study 2 (n=4) with squares. Individual animals within the studies can be tracked with the different filling of the symbols **(B)** Bacterial burden in the urine was quantified prior to challenge on day 0 and on days 2, 7, and 14 post-challenge in Study 3. This study consisted of 2 groups, with group 1 (circles, n=4) going to 14 days post-challenge and group 2 (squares, n=4) going up to 7 days post-challenge. Individual animals within a group can be tracked with the different filling of the symbols. The mean of each group is indicated with a black line. LOD, Limit of detection.

**Figure 4:**
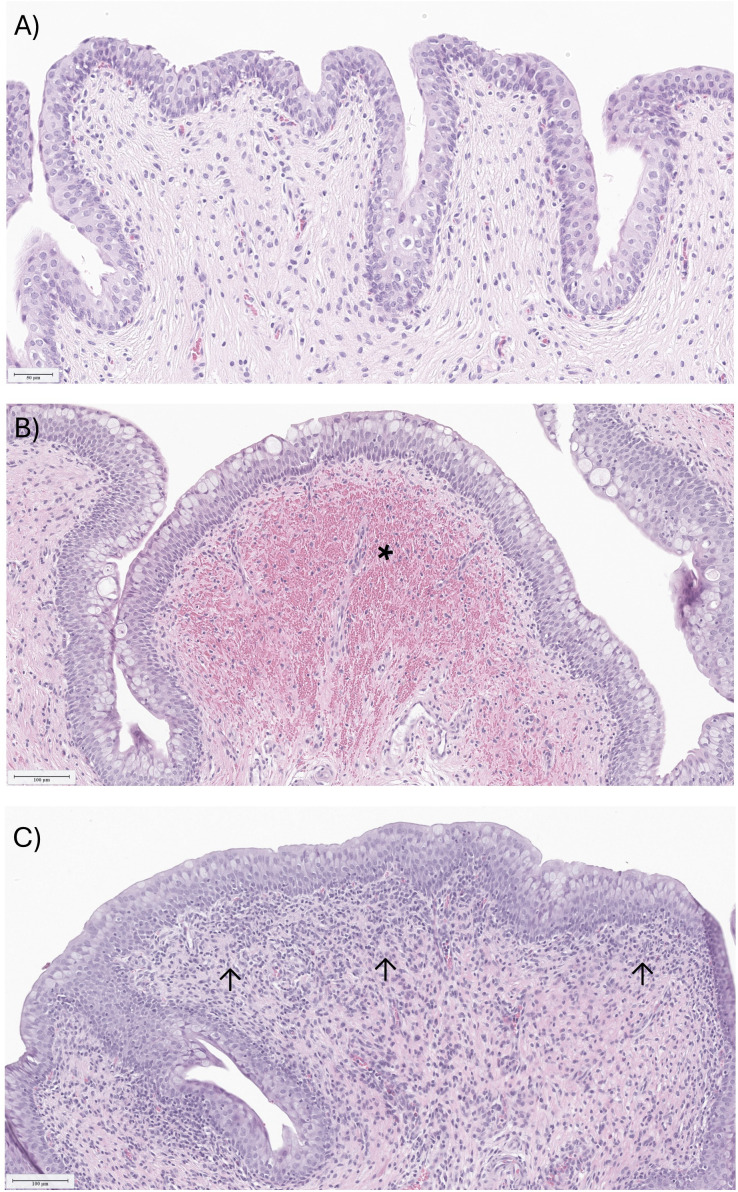
Representative microscopic images of the urinary bladder mucosa of a minipig (HE: 20x) from study 3. **(A)** non-inflamed bladder mucosa. **(B)** Bladder with subepithelial hemorrhage (depicted with a *) and minimal mucinous metaplasia of urothelium in an animal 7 days after challenge. **(C)** Bladder showing chronic inflammation in the lamina propria (depicted with arrows) in an animal 14 days after challenge.

In Study 1, we identified an inoculum concentration inducing an efficacious bacterial load in the urine while avoiding severe lesions in the urinary tract. Hereafter, we set out to investigate the duration of infection for this UTI minipig model. An independent experiment was performed with a duration of 28 days using an inoculum of approximately 7 log_10_ CFU of UPEC strain UTI89 (Study 2, **Supplementary Table 1**). These results were analyzed next to data from Group 3 Study 1, which also had a duration of 28 days using an inoculum of approximately 7 log10 CFU of UPEC strain UTI89. Between both studies (n=8), only one animal exhibited pre-existing bacteria in the urine while all other animals exhibited urine sterility on day 0 (**Figure 3A**). On day 2, urine CFU confirmed establishment of infection in all animals of both studies following challenge. By day 14, the infection prevalence was 75% (n=6/8) over both studies (**Figure 3A**), which we considered an adequate infection rate for minipig infection studies. On day 28, only 37.5% (n=2/8) of the animals still had detectable bacterial load in the urine. This indicated that a large number of animals naturally resolved their UTI infections by day 28. Therefore, we investigated day 7 and day 14 as feasible endpoints for our minipig UTI model while maintaining the same UTI89 inoculum (Study 3, **Supplementary Table 1**). On day 7 post-challenge, 87.5% (n = 7/8) of the animals had quantifiable bacteria in the urine. Of the animals that were kept until day 14, 75% (n=3/4) remained positive for bacteria in the urine (**Figure 3B**). Histopathological analysis indicated that shorter infection duration (day 7) was associated with more severe bladder hemorrhages, whereas longer duration (day 14) corresponded with higher incidence and severity of bladder inflammation (**Supplementary Table 3**). Representative microscopic images of non-inflamed bladder mucosa, subepithelial bladder hemorrhages, and bladder inflammation are depicted in **Figure 4**. In conclusion, the optimal duration to detect infection in the minipig UTI model, taking into account bacterial load in the urine and histopathology of the urinary tract, was 14 days.

### 3.3 Biomarkers in the UTI minipig model

**Figure 5:**
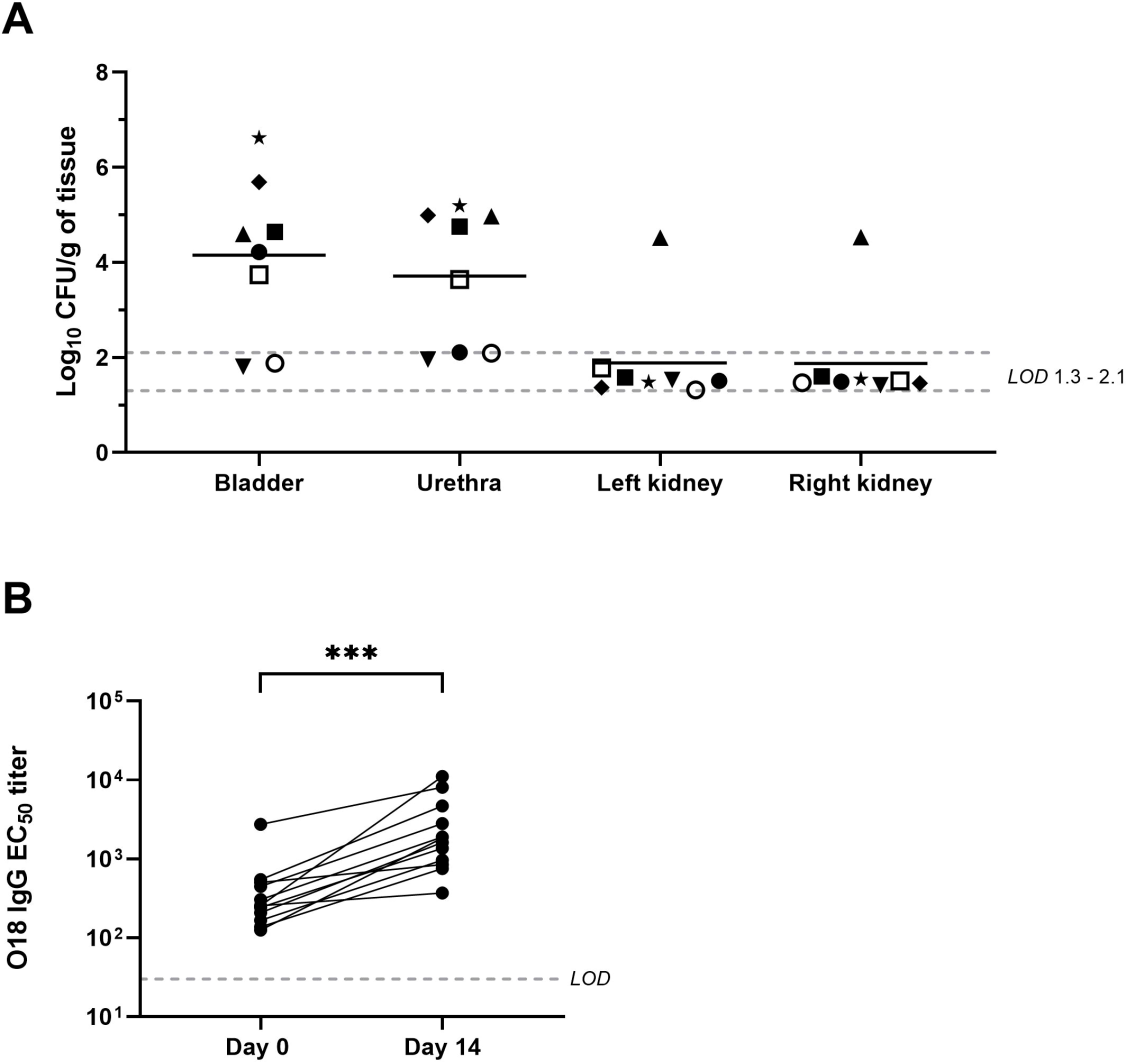
Bacterial load and O18 antibodies **(A)** bacterial quantification in bladder, urethra, left and right kidney of minipigs (n=8, each animal indicated with a different symbol) challenged with approximately 7 log_10_ CFU UTI89 in Study 4. Bacterial burden was quantified on day 14 post-challenge. The mean over the whole group is indicated with a black line. LOD = limit of detection. No statistics were performed. **(B)** O18 specific IgG antibodies in serum of minipigs from study 4. Antibodies were detected with ELISA and EC50 titers are shown per animal for day 0 prior to challenge and day 14 post-challenge connected with a black line. The LOD was 30 EC50 and indicated with a dotted line. Wilcoxon matched pairs signed rank test for non-parametric paired samples was performed comparing day 14 with day 0. ***P<0.001.

To further study the UTI minipig model and consider other parameters that could verify infection, we conducted an additional study, Study 4 (**Supplementary Table 1**), with an endpoint of 14 days post-challenge. In this study, we performed an in-depth analysis of the bacterial load in different tissues and assessed serum antibodies before challenge and during infection. The urine bacterial load seen in this study was comparable to previous studies with a similar inoculum of UTI89 (approximately 7 log_10_ CFU) (**Supplementary Figure 2**). One animal from this study exhibited a pre-existing bacterial infection of approximately 3 log_10_ CFU/mL in the urine collected just before challenge (day 0), with what was later determined to be a different *E. coli* serotype (mixed O2/O110) than the challenge strain (O18). However, this animal was already challenged with UTI89 by the time this was known due to the prolonged nature of plating, culturing, and reading of the urine plates. Therefore, this animal remained in the study and showed a detectable bacterial load in the urine on days 2, 7, and 14 post-challenge, comparable with the other animals. Bacterial burden in the tissues on day 14 was enumerated with 75% (n=6/8) of the animals showing bacteria in the bladder and 62.5% (n=5/8) of the animals presenting with bacteria in the urethra (**Figure 5A**). No clear progression was visible to pyelonephritis in this study as only one animal showed bacteria in the kidneys. However, upon histopathological examination, no signs of pyelonephritis were detected in this animal. Another animal from this study showed chronic-active inflammation with fibrosis of the kidneys extending into the renal cortex, but no bacterial load was detected in the kidneys of this animal. Histopathological evaluation of the other tissues like the bladder did confirm the tissue CFU findings (**Supplementary Table 4**).

To evaluate the development of strain-specific antibodies in reaction to infection with UTI89, serum antibodies against the O18 antigen were measured by ELISA. Preexisting O18 IgG antibodies were detected in minipig serum collected on day 0 before the intraurethral inoculation in all animals (**Figure 5B**). These O18 IgG antibody titers increased significantly (p< 0.001) after intraurethral inoculation with UTI89 in Study 4, revealing a systemic immune reaction in minipigs infected using our UTI model.

To analyze the susceptibility of the minipig UTI model to infection, a small pilot mock infection study was conducted in which the animals received intraurethral inoculation with sterile saline at day 0. Despite the absence of intentional bacterial challenge, microbiological analysis revealed that one animal tested positive for *Proteus mirabilis* at day 2, while the second animal was positive for *E. coli* with serotype O25A from day 2 through day 14 (**Supplementary Figure 3**). These findings suggest that the minipig UTI model is sensitive to bladder infections or colonization. The detected infections may have been present in the bladder at baseline below the detection limit of the urine bacterial load quantification assay or alternatively may have been introduced during handling or sampling procedures despite the stringent measures taken to minimize contamination.

### 3.4 Recovery strain identification is key to understanding the infection in the UTI minipig model

To assess whether the UTI89 challenge strain was also the strain seen on plated urine from days 2 and 7 post-challenge (Study 4, **Supplementary Table 1**), two colonies from each plate were isolated and used to check hemolytic capacity by plating on blood agar plates, tested with an agglutination assay for agglutination to *E. coli* antisera, and evaluated using WGS for specific O serotype, H type and capsule type. UPEC strain UTI89 forms hemolytic colonies on blood agar plates (26). Hemolysis testing of colonies from day 2 and day 7 post-challenge plated urine revealed that only one animal tested positive for non-hemolytic colonies on both days 2 and 7 post-challenge (**Table 1**). A different animal displayed hemolytic colonies on day 2 but presented with non-hemolytic colonies on day 7 post-challenge. All other animals expressed hemolytic colonies on the blood agar plates, pointing towards the presence of the UTI89 strain in the urine.

**Table 1:** Hemolytic capacity of bacterial colonies on blood agar plates from study 4.

Animal	Day 2	Day 7
1262394	Hemolytic	Hemolytic
1262556	Hemolytic	Hemolytic
1262351	Hemolytic	Hemolytic
1262491	Non hemolytic	Non hemolytic
1262696	Hemolytic	Non hemolytic
1262891	Hemolytic	Hemolytic
1262769	Hemolytic	Hemolytic
1262840	Hemolytic	Hemolytic

Minipig urine isolates from day 2 and 7 post-challenge were plated on blood agar plates and examined for hemolysis around the colonies. Observed hemolysis is stated per animal (n=8) per day.

**Figure 6:**
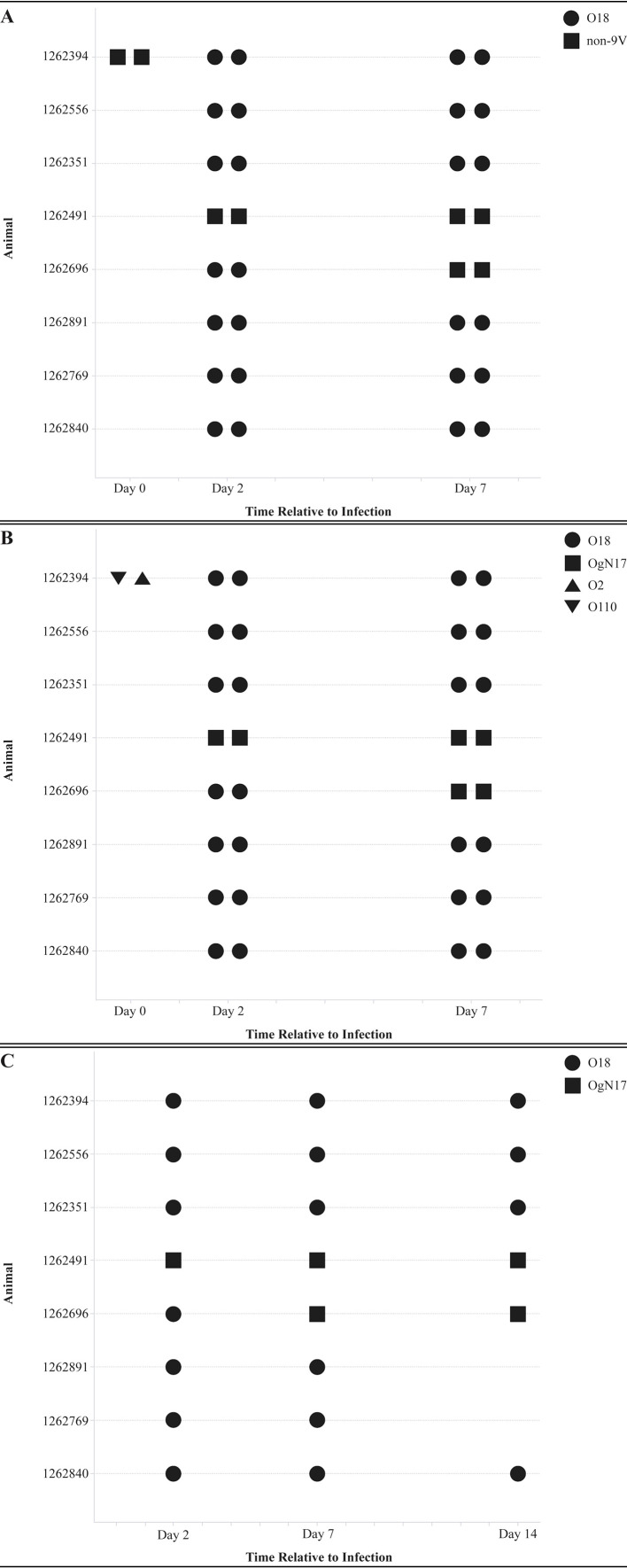
Agglutination and whole genome sequencing (WGS) based serotype determination of bacterial isolates picked from MacConkey agar CFU count plates in study 4. **(A)** Minipig urine isolates from day 2 and 7 post-challenge were tested in Agglutination to the nine (9V) most common human bacteremia O serotypes (O1, O2, O4, O6, O15, O16, O18, O25, and O75) was performed and stated as positive for a serotype (O18 agglutination depicted as circles) or not positive for all nine serotypes tested (non-9V, depicted as squares). No symbol is depicted on the timepoint when no colonies were detected in urine that could be isolated. **(B)** Minipig urine isolates from day 2 and 7 post-challenge were analyzed with WGS for serotype identification based on *wzy*, *wzx*, *wzt*, and *wzm* genes with the O-genotyper application. O18 serotype based on WGS is depicted with a circle, OgN17 serotype with a square, O110 with an upward triangle, and O2 with a downward triangle. Serotypes based on WGS are stated per animal (n=8) per timepoint. No symbol is depicted on the timepoint when no colonies were detected in urine that could be isolated. **(C)** Plate scrapes from minipig urine of day 2, 7, and 14 post-challenge were analyzed with WGS for serotype identification based on *wzy*, *wzx*, *wzt*, and *wzm* genes with the O-genotyper application. O18 serotype based on WGS is depicted with a circle and OgN17 serotype with a square. Serotypes based on WGS are stated per animal (n=8) per timepoint. No symbol is depicted on the timepoint when no colonies were detected in urine that could be isolated.

However, hemolytic colonies alone do not confirm if the strain isolated from the urine matches the challenge strain. O serotype determination with an agglutination assay can provide more information on the presence of the correct strain. To this end, agglutination assays were performed for the nine (9V) most common human bacteremia O serotypes (O1, O2, O4, O6, O15, O16, O18, O25, and O75). This revealed that the two animals presenting with non-hemolytic colonies also did not agglutinate to the nine O serotypes, confirming that these colonies were not the UTI89 strain (O18). All animals with hemolytic colonies showed agglutination to the O18 serotype (**Figure 6A**).

Further confirmation of the strain serotypes, including H type and capsule type, was performed with WGS on bacterial isolates from day 2 and 7 that were used for agglutination and hemolytic capacity screening. The animals that presented with non-hemolytic colonies and no agglutination to the nine O serotype antisera also showed with WGS that the strain was not an O18 serotype, but an OgN17 serotype (**Figure 6B**). This serotype is a distinct genotype from O18 with approximately 40% identical *wzx* and *wzy* genes. The OgN17 is determined as an ST602 strain with H21 and group 1 CL35 capsule types (**Supplementary Table 5**). For the other 6 animals, it was confirmed with WGS that the isolates derived from the urine at day 2 and day 7 was an O18 serotype. The different isolates derived per animal showed a homogenous result and therefore, plate scrapes were analyzed for day 14. This revealed that the two animals with the OgN17 serotype *E. coli* strain remained infected with this strain and the other animals remained infected with the O18 strain at day 14 (**Figure 6C**). The O18 strain isolated from the urine isolates and scrapes had a ST95 multilocus sequence typing (MLST) with an H7 and G2_KL8_A1-K1 capsule type (**Table 2**). This is identical to the strain that was used for the inoculum, as one of the challenge glycerol stocks was tested with WGS and came back as O18 serotype with ST95 (**Table 2**). Therefore, it could be concluded that the challenge strain was detected in urine samples of most of the animals for the duration of the experiment.

**Table 2:** Whole genome sequencing based serotypes for isolates picked from MacConkey agar CFU count plates for each animal at each time point for study 4.

Animal	Day	ST	O serotype	H type	K capsule type	FimH type
Inoculum	NA	95	O18	H7	G2_KL8A1_K1	18
1262394	2	95	O18	H7	G2_KL8A1_K1	18
1262394	7	95	O18	H7	G2_KL8A1_K1	18
1262394	14	95	O18	H7	G2_KL8A1_K1	18
1262556	2	95	O18	H7	G2_KL8A1_K1	18
1262556	7	95	O18	H7	G2_KL8A1_K1	18
1262556	14	95	O18	H7	G2_KL8A1_K1	18
1262351	2	95	O18	H7	G2_KL8A1_K1	18
1262351	7	95	O18	H7	G2_KL8A1_K1	18
1262351	14	95	O18	H7	G2_KL8A1_K1	18
1262491	2	602	OgN17	H21	group1_CL35_	86
1262491	7	602	OgN17	H21	group1_CL35_	86
1262491	14	602	OgN17	H21	group1_CL35_	86
1262696	2	95	O18	H7	G2_KL8A1_K1	18
1262696	7	602	OgN17	H21	group1_CL35_	86
1262696	14	602	OgN17	H21	group1_CL35_	86
1262891	2	95	O18	H7	G2_KL8A1_K1	18
1262891	7	95	O18	H7	G2_KL8A1_K1	18
1262891	14	–	–	–	–	–
1262769	2	95	O18	H7	G2_KL8A1_K1	18
1262769	7	95	O18	H7	G2_KL8A1_K1	18
1262769	14	-	-	-	-	-
1262840	2	95	O18	H7	G2_KL8A1_K1	18
1262840	7	95	O18	H7	G2_KL8A1_K1	18
1262840	14	95	O18	H7	G2_KL8A1_K1	18

ST, sequence type; NA, not applicable.

Overall, we were able to confirm that a majority of minipigs inoculated in our UTI model presented with the challenge strain, UPEC strain UTI89, in the urine, however, it was necessary to use WGS to determine this as we found evidence of a different recovered strain in 25% of the animals.

## 4 Discussion

In this paper we aimed to establish and optimize a UPEC UTI minipig model using a more natural route of infection through intraurethral inoculation. The US Food and Drug Administration and European Medicine Agency guidelines consider a bacterial load of 5 log_10_ CFU/mL in the urine as the threshold for UTIs (27). Minipigs and humans share a similar bladder size and urine voiding force; therefore, we hypothesized that the human clinical bacterial load would be an appropriate threshold for the minipigs. Using our optimized model, we found similar bacterial loads in the urine of minipigs challenged with UPEC strain UTI89 comparable to the human clinical bacterial load threshold. The best inoculum concentration for consistent infection was found to be approximately 7 log_10_ CFU per minipig. Due to the relatively small sample size, this study would benefit from replication with larger group sizes to strengthen these findings. With this inoculum concentration, all minipigs showed infection with bacteria in the urine on day 2, with most maintaining infection up to 14 days. From 14 days onwards, analysis of the bacterial load in urine showed consistent natural clearance of the infections. An experiment with another UPEC strain of the O25b serotype with the same inoculum concentration resulted in a steady infection out to day 14 (data not shown), showing that this model has the potential to support other strains. Similar UTI studies using a farm pig model, with an inoculum delivered directly into the bladder, resulted in observable infection in the urine up to 23 days when challenged with 10 log_10_ CFU UTI89 per pig (12), while a lower inoculum of 4 log_10_ CFU per pig resulted in some natural clearance on day 14 (28). This highlights the necessity of testing different inoculum concentrations when using different models to establish a consistent infection.

While urine bacterial concentrations of UPEC UTIs are similar in range between humans and pigs, there are differences in clinical UTI disease presentation. In humans, pain and straining to urinate are hallmarks of the disease (29, 30); this is more difficult to observe in pigs as they are prey animals. Video surveillance of pigs may be helpful in assessing these parameters, but additional biomarkers should be investigated. Another similarity between humans and pigs is the presence of O serotype antibodies in the serum of both human and minipigs. These antibodies are detectable in the general human population (31) and indicate prior exposure to *E. coli.* In this study, we specifically examined antibodies against the O18 O antigen, as UTI89 is an O18 positive strain. Preexisting O18 specific antibodies were detected at day 0 in minipig serum. Following challenge with the UPEC UTI89 strain, an increase in O18 specific antibody levels was observed by day 14, indicating that the immune response was boosted by the UTI89 infection. Clinical chemistry and hematology were also measured in urine and serum of challenged minipigs, but no significant or consistent correlations with infection were detected throughout the course of the study (data not shown). Earlier timepoints for urine analyses might reveal changes in urine composition. However, these tests are largely limited by exogenous factors such as hydration, diet, and estrus cycle, so they may not serve as consistent biomarkers of infection.

Histopathologic analysis of the tissues revealed signs of infection in tissues such as bladder and urethra of the minipigs challenged with UTI89, correlating with the bacterial load seen in these tissues. Remarkably, we saw minimal dissemination towards the kidneys with the 7 log_10_ CFU inoculum on days 14 and 28. This was unexpected as the UTI89 strain is a human cystitis strain (32) and we expected to see kidney dissemination. Use of a higher inoculum dose resulted in observable histopathological findings in the kidneys on day 28, even in the absence of quantifiable bacteria in the urine. This might point to a dose-dependent requirement for dissemination. A longer interval between the initial bladder infection and tissue bacterial quantification may contribute to an increased rate of kidney infection. Another explanation of the low kidney dissemination could be that the UTI89 strain is highly dependent on type 1 fimbriae for adherence in the bladder compared to the kidneys, where P fimbriae expression is essential (33).

In our minipig UTI model, the UTI89 strain used for the initial challenge was not consistently recovered from the urine samples collected throughout the study, suggesting that a secondary infection with a different UPEC strain took place. This was most noticeable in the hemolytic capacity of the bacteria on the blood agar plates from hemolytic to non-hemolytic colonies, indicating a different strain was present. This observation was further supported by negative results while testing for agglutination to the nine most common *E. coli* bacteremia O serotype antisera, including the O18 positive UTI89 strain. Identification of the strains in urine of each animal with WGS to determine not only O serotype, but also sequence type, H type, K capsule type, and FimH type, was used to provide greater confidence that the recovered strains from urine were comparable to the challenge strain. In 25% of the animals from Study 4, the recovery strain was different from the challenge strain. Additionally, no differences in urine bacterial loads or histopathological findings were found between animals where the UTI89 strain versus an alternative strain was recovered. This shows the importance of strain identification for interpreting experimental challenge outcomes and that quantification of bacterial load is not sufficient to support conclusions on infection status.

Our results highlight the need for caution when interpreting outcomes from this and other UTI models, as the strain recovered from urine is not always identical to the challenge strain. Careful verification of isolate identity is, therefore, essential to ensure accurate evaluation of intervention effects with vaccine and antibiotic studies. Overall, we developed a novel minipig UTI model using an intraurethral challenge with UPEC strain UTI89, providing a valuable model for assessing therapeutic efficacy of vaccines or antibiotics and improving translation of preclinical findings to the human clinical setting.

## Data Availability

The datasets presented in this study can be found in online repositories. The names of the repository/repositories and accession number(s) can be found in the article/**Supplementary Material**.
