# Supplementary material for: Establishment and optimization of an E. coli urinary tract infection model in Göttingen minipigs with strain recovery and characterization
Source: Front Immunol. 2026 May 18;17:1842934. doi: 10.3389/fimmu.2026.1842934 (PMC13223159; doi:10.3389/fimmu.2026.1842934)
Supplement: Supplementary file 1 [file DataSheet1.docx]

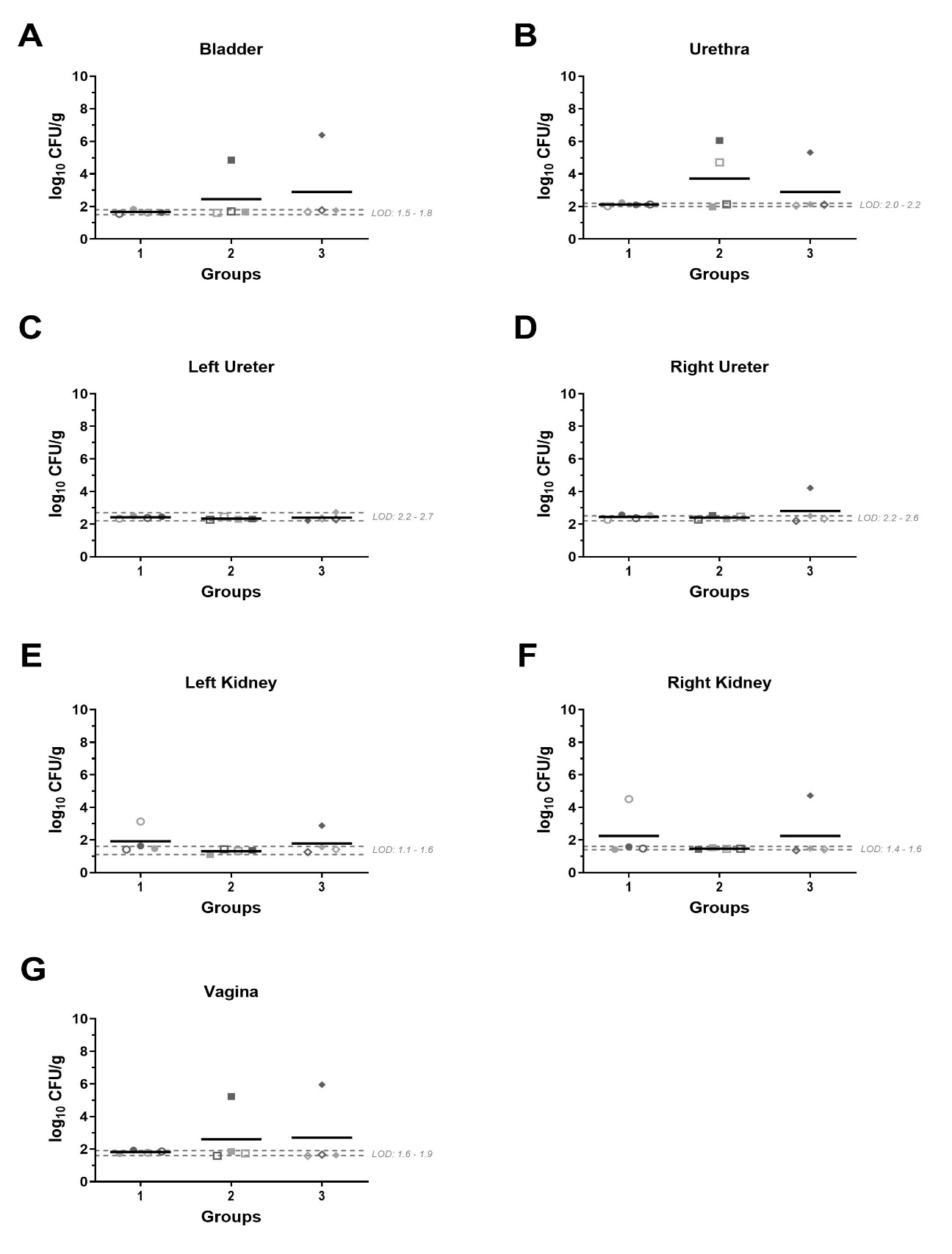


**Supplementary Figure 1**. Bacterial quantification in the tissues on Day 28 of minipigs challenged with different amounts of UPEC strain UTI89 (Study 1). The high CFU group (circles, n=4) received approximately 9 log10 CFU (8.80-9.05 log10 CFU), the mid CFU group (squares, n=4) received approximately 8 log10 CFU (7.80-8.05 log10 CFU), and the low CFU group (diamonds, n=4) received approximately 7 log10 CFU (6.80-7.05 log10 CFU). Individual animals within a group can be tracked with the different filling of the symbols. Mean of each group is indicated with a black line. LOD: Limit of detection. **A)** Bladder **B)** Urethra **C)** Left Ureter **D)** Right Ureter **E)** Left Kidney **F)** Right Kidney **G)** Vagina
