# Supplementary material for: Establishment and optimization of an E. coli urinary tract infection model in Göttingen minipigs with strain recovery and characterization
Source: Front Immunol. 2026 May 18;17:1842934. doi: 10.3389/fimmu.2026.1842934 (PMC13223159; doi:10.3389/fimmu.2026.1842934)

Supplementary Material

# Supplementary Data

**Supplementary Figure 2.** Bacterial quantification in urine of minipigs (N=8) challenged with approximately 7 log_10_ CFU UTI89 in Study 4. Bacterial burden in the urine was quantified prior to challenge on day 0 and on days 2, 7, and 14 post-challenge. Mean of each group is indicated with a black line. LOD = Limit of detection


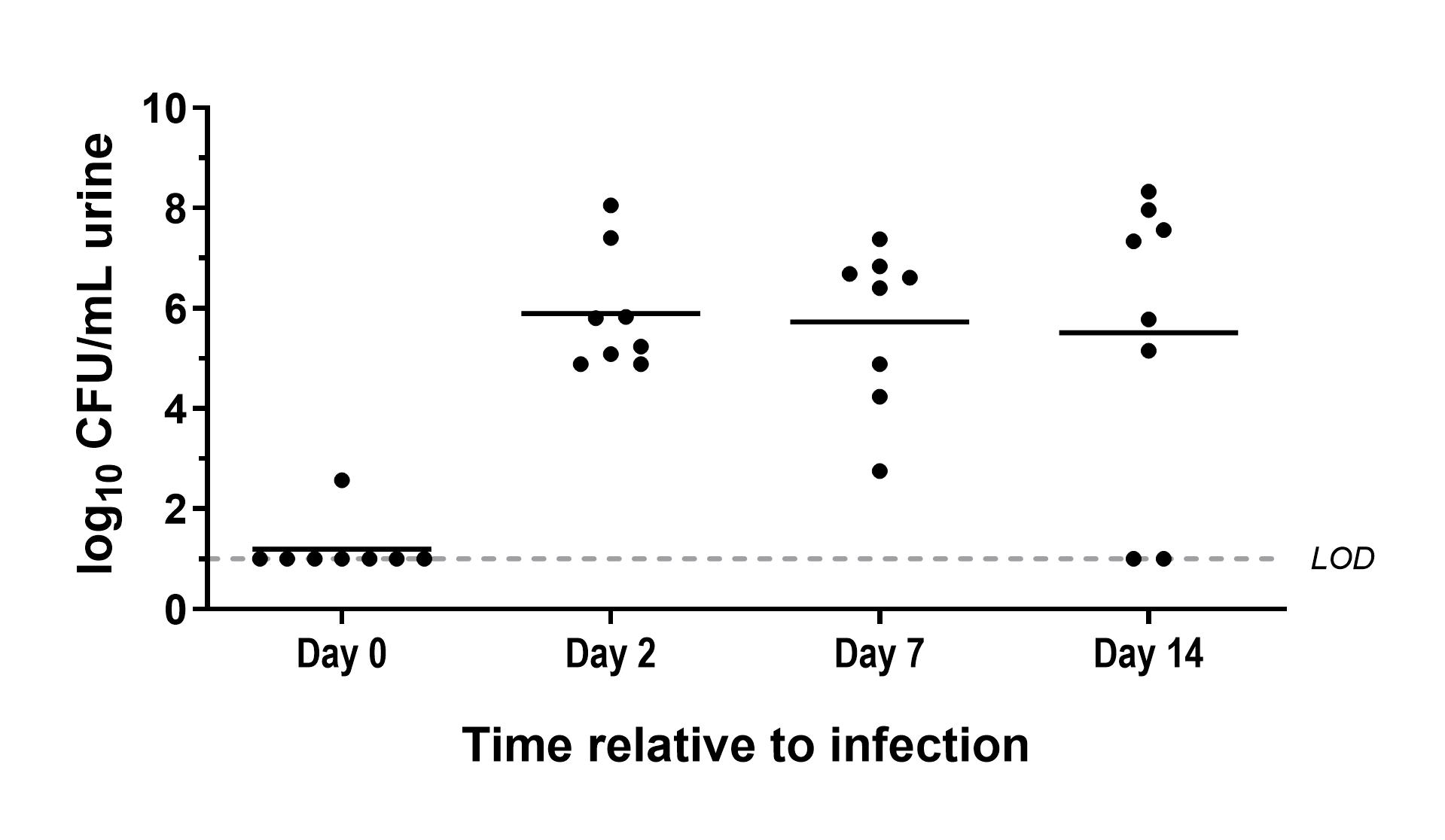

Supplement: Supplementary file 2 [file DataSheet2.docx]
