# Supplementary material for: Establishment and optimization of an E. coli urinary tract infection model in Göttingen minipigs with strain recovery and characterization
Source: Front Immunol. 2026 May 18;17:1842934. doi: 10.3389/fimmu.2026.1842934 (PMC13223159; doi:10.3389/fimmu.2026.1842934)
Supplement: Supplementary file 3 [file DataSheet3.docx]

**Supplementary Figure 3.** Bacterial quantification in urine of minipigs (N=2) inoculated with sterile saline in a pilot mock infection study. Bacterial burden in the urine was quantified prior to challenge on day 0 and on days 2, 7, and 14 post-challenge. Animal 1, indicated with a circle, was positive for *E. coli* serotype O25A. Animal 2, indicated with a square, was positive for *Proteus mirabilis* on day 2. LOD = Limit of detection
