# Supplementary material for: Establishment and optimization of an E. coli urinary tract infection model in Göttingen minipigs with strain recovery and characterization
Source: Front Immunol. 2026 May 18;17:1842934. doi: 10.3389/fimmu.2026.1842934 (PMC13223159; doi:10.3389/fimmu.2026.1842934)
Supplement: Supplementary file 4 [file Table1.docx]

**Supplementary Table 1**: List of Studies described in the manuscript with group allocation, inoculum concentration, and urine collection timepoints.

| **Study Name** | **Study Description** | **Groups** | **Number of Animals** | **Challenge Strain UTI89*** | **Urine Collection Timepoints** |
| --- | --- | --- | --- | --- | --- |
| **Study 1** | Inoculum Titration | 1: High CFU | 4 | 9.05 log10 (n=2)  8.80 log10 (n=2) | D0, D2, D7, D14, D21, D28 |
|  |  | 2: Mid CFU | 4 | 8.05 log_10_ (n=2)  7.80 log_10_ (n=2) |  |
|  |  | 3: Low CFU | 4 | 7.05 log_10_ (n=2)  6.80 log_10_ (n=2) |  |
| **Study 2** | Duration of Infection | N/A | 4 | 6.45 log_10_ | D0, D2, D7, D14, D23, D28 |
| **Study 3** | Duration of Infection | 1: D14 Necropsy | 4 | 7.60 log_10_ | D0, D2, D7, D14 |
|  |  | 2: D7 Necropsy | 4 | 7.40 log_10_ | D0, D2, D7 |
| **Study 4** | Biomarker & WGS Analysis | N/A | 8 | 7.17 log_10_ (n=4) 7.26 log_10_ (n=4) | D0, D2, D7, D14 |
| **Pilot mock study** | Mock infection study | N/A | 2 | Sterile saline | D0, D2, D7, D14 |

*Small differences in inoculum concentrations in the same groups are due to challenge replication on a different day.
