# Supplementary material for: Establishment and optimization of an E. coli urinary tract infection model in Göttingen minipigs with strain recovery and characterization
Source: Front Immunol. 2026 May 18;17:1842934. doi: 10.3389/fimmu.2026.1842934 (PMC13223159; doi:10.3389/fimmu.2026.1842934)
Supplement: Supplementary file 5 [file Table2.docx]

**Supplementary Table 2:** Histopathology incidences and severity grades of urinary tract tissue in minipigs challenged on day 0 with different amounts of UPEC strain UTI89 in Study 1. Tissues were collected at necropsy day 28. The high CFU group (n=4) received approximately 9 log_10_ CFU, the mid CFU group (n=4) received approximately 8 log_10_ CFU, and the low CFU group (n=4) received approximately 7 log_10_ CFU. Data is presented as incidence (the number of animals afflicted) per grade. Grade 1: minimal histological change, grade 2: mild, grade 3: moderate, grade 4: marked, or grade 5: severe.

|  |  |  | **Group 1 High CFU** | **Group 2 Mid CFU** | **Group 3 Low CFU** |
| --- | --- | --- | --- | --- | --- |
|  | Number of Animals |  | 4 | 4 | 4 |
| Urinary Bladder  (dorsal) | hemorrhage | grade 2 | 1 | 0 | 0 |
|  | mucous metaplasia | grade 1 | 1 | 1 | 0 |
|  |  | grade 3 | 3 | 1 | 1 |
|  | inflammation | grade 1 | 0 | 0 | 2 |
|  |  | grade 2 | 1 | 2 | 0 |
|  |  | grade 3 | 2 | 1 | 0 |
|  |  | grade 4 | 1 | 0 | 1 |
|  | edema | grade 2 | 1 | 0 | 0 |
|  |  | grade 4 | 1 | 0 | 0 |
| Urethra | congestion | grade 4 | 0 | 1 | 0 |
|  | inflammation | grade 3 | 4 | 2 | 1 |
|  |  | grade 4 | 0 | 1 | 0 |
| Ureter (left) | inflammation | grade 1 | 1 | 1 | 0 |
| Ureter (right) | inflammation | grade 1 | 1 | 1 | 0 |
| Vagina | inflammation | grade 1 | 1 | 0 | 0 |
|  |  | grade 2 | 0 | 1 | 0 |
| Kidney  (left) | glomerulonephritis | grade3 | 1 | 0 | 0 |
|  | inflammation tubulointerstitial | grade 3 | 0 | 0 | 1 |
|  |  | grade 4 | 1 | 0 | 0 |
|  | pyelonephritis | grade 3 | 1 | 1 | 0 |
|  | degeneration | grade 1 | 1 | 1 | 0 |
|  | inflammation | grade 1 | 0 | 1 | 0 |
|  | mucous metaplasia | grade 3 | 1 | 0 | 0 |
| Kidney  (right) | glomerulonephritis | grade 1 | 0 | 1 | 0 |
|  |  | grade3 | 1 | 0 | 0 |
|  | inflammation tubulointerstitial | grade 2 | 0 | 1 | 0 |
|  |  | grade 3 | 1 | 0 | 1 |
|  | pyelonephritis | grade 3 | 1 | 0 | 0 |
|  | degeneration | grade 1 | 2 | 1 | 0 |
|  | inflammation | grade 1 | 0 | 0 | 1 |
