# Supplementary material for: Establishment and optimization of an E. coli urinary tract infection model in Göttingen minipigs with strain recovery and characterization
Source: Front Immunol. 2026 May 18;17:1842934. doi: 10.3389/fimmu.2026.1842934 (PMC13223159; doi:10.3389/fimmu.2026.1842934)
Supplement: Supplementary file 6 [file Table3.docx]

**Supplementary Table 3:** Histopathology incidences and severity grades of urinary tract tissue in minipigs challenged on day 0 with approximately 7 log_10_ CFU UPEC strain UTI89 in Study 3. Tissues were collected at necropsy on day 14 (group 1) or day 7 (group 2). Data is presented as incidence (the number of animals afflicted) per grade. Grade 1: minimal histological change, grade 2: mild, grade 3: moderate, grade 4: marked, or grade 5: severe.

|  |  |  | **Group 1 14 days** | **Group 2 7 days** |
| --- | --- | --- | --- | --- |
|  | Number of Animals |  | 4 | 4 |
| Urinary  Bladder  (ventral and dorsal combined) | hemorrhage | grade 1 | 3 | 0 |
|  |  | grade 3 | 0 | 2 |
|  | mucous metaplasia | grade 2 | 2 | 2 |
|  |  | grade 3 | 2 | 2 |
|  | inflammation | grade 1 | 0 | 2 |
|  |  | grade 2 | 2 | 1 |
|  |  | grade 3 | 2 | 0 |
|  | edema | grade 2 | 3 | 3 |
|  |  | grade 3 | 1 | 1 |
|  | degeneration | grade 2 | 0 | 1 |
|  | necrosis/inflammation vascular | grade 1 | 0 | 1 |
| Urethra | congestion | grade 1 | 0 | 1 |
|  |  | grade 2 | 0 | 1 |
|  | infiltrate | grade 1 | 2 | 2 |
|  |  | grade 2 | 1 | 1 |
|  | edema | grade 1 | 1 | 0 |
|  | metaplasia | grade 2 | 1 | 0 |
| Ureter (left) | metaplasia | grade 1 | 0 | 1 |
|  | infiltrate | grade 1 | 1 | 1 |
| Ureter (right) | metaplasia | grade 1 | 0 | 1 |
|  | infiltrate | grade 1 | 0 | 1 |
| Vagina | no findings |  | 0 | 0 |
| Kidney  (left) | glomerulosclerosis | grade 2 | 0 | 1 |
|  | infiltrate | grade 1 | 2 | 0 |
|  | dilation | grade 1 | 1 | 1 |
|  | fibrosis | grade 2 | 0 | 1 |
| Kidney  (right) | glomerulosclerosis | grade 2 | 0 | 1 |
|  | inflammation | grade 2 | 1 | 0 |
|  | dilation | grade 1 | 1 | 1 |
|  | fibrosis | grade 2 | 0 | 1 |
