# Supplementary material for: Establishment and optimization of an E. coli urinary tract infection model in Göttingen minipigs with strain recovery and characterization
Source: Front Immunol. 2026 May 18;17:1842934. doi: 10.3389/fimmu.2026.1842934 (PMC13223159; doi:10.3389/fimmu.2026.1842934)
Supplement: Supplementary file 7 [file Table4.docx]

**Supplementary Table 4:** Histopathology incidences and severity grades of urinary tract tissue in minipigs challenged on day 0 with approximately 7 log_10_ CFU UPEC strain UTI89 in Study 4. Tissues were collected at necropsy day 14. Data is presented as incidence (the number of animals afflicted) per grade. Grade 1: minimal histological change, grade 2: mild, grade 3: moderate, grade 4: marked, or grade 5: severe.

|  | Number of Animals |  | 8 |
| --- | --- | --- | --- |
| Urinary Bladder  (dorsal)* | hemorrhage | grade 1 | 1 |
|  |  | grade 2 | 1 |
|  | mucous metaplasia | grade 1 | 3 |
|  |  | grade 2 | 2 |
|  |  | grade 3 | 1 |
|  | inflammation | grade 1 | 1 |
|  |  | grade 2 | 4 |
|  |  | grade 3 | 2 |
|  | edema | grade 1 | 1 |
|  |  | grade 2 | 6 |
|  | infiltrate | grade 1 | 1 |
|  |  | grade 2 | 1 |
| Urinary Bladder  (ventral) | hemorrhage | grade 1 | 1 |
|  |  | grade 2 | 2 |
|  |  | grade 3 | 1 |
|  | mucous metaplasia | grade 1 | 3 |
|  |  | grade 2 | 3 |
|  |  | grade 3 | 2 |
|  | inflammation | grade 1 | 3 |
|  |  | grade 2 | 4 |
|  |  | grade 3 | 1 |
|  | edema | grade 1 | 2 |
|  |  | grade 2 | 4 |
|  |  | grade 3 | 2 |
|  | infiltrate | grade 1 | 2 |
|  |  | grade 2 | 2 |
| Urethra | inflammation | grade 1 | 1 |
|  | infiltrate | grade 1 | 3 |
|  |  | grade 2 | 1 |
|  |  | grade 3 | 1 |
| Ureter (left) | metaplasia | grade 1 | 2 |
|  | infiltrate | grade 1 | 1 |
| Ureter (right) | metaplasia | grade 1 | 2 |
| Kidney  (left) | infiltrate | grade 1 | 1 |
|  | fibrosis | grade 3 | 1 |
|  | dilation | grade 2 | 1 |
|  | inflammation | grade 3 | 1 |
|  | metaplasia | grade 1 | 2 |
|  |  | grade 3 | 1 |
| Kidney  (right) | infiltrate | grade 1 | 1 |
|  | fibrosis | grade 3 | 1 |
|  | inflammation | grade 4 | 1 |
|  | metaplasia | grade 2 | 1 |

* In the urinary bladder (dorsal) only 7 animals were graded instead of 8 animals.
