# Supplementary material for: Establishment and optimization of an E. coli urinary tract infection model in Göttingen minipigs with strain recovery and characterization
Source: Front Immunol. 2026 May 18;17:1842934. doi: 10.3389/fimmu.2026.1842934 (PMC13223159; doi:10.3389/fimmu.2026.1842934)
Supplement: Supplementary file 8 [file Table5.docx]

**Supplementary Table 5**: Agglutination and whole genome sequencing based serotypes for isolates picked from MacConkey agar CFU count plates for each animal at each time point for Study 4. Agglutination to the nine (9V) most common human bacteremia O serotypes (O1, O2, O4, O6, O15, O16, O18, O25, and O75) was performed and stated as positive for a serotype or not positive for all nine serotypes tested (non-9V). Blood agar plates were used for hemolytic capacity observations. ST = sequence type.

| Animal | Day | Blood Agar Observations | Agglutination Serotype | ST | O serotype | H type | K capsule type | FimH type |
| --- | --- | --- | --- | --- | --- | --- | --- | --- |
| 1262394 | 0 | Non-Hemolytic | non-9V | 348 | O110 | H28 | group1_CL35_ | 235 |
| 1262394 | 0 | Hemolytic; Mucoid | non-9V | 141 | O2 | H6 | group1_CL35_ | 5 |
| 1262394 | 2 | Hemolytic | O18 | 95 | O18 | H7 | G2_KL8A1_K1 | 18 |
| 1262394 | 2 | Hemolytic | O18 | 95 | O18 | H7 | G2_KL8A1_K1 | 18 |
| 1262394 | 7 | Hemolytic | O18 | 95 | O18 | H7 | G2_KL8A1_K1 | 18 |
| 1262394 | 7 | Hemolytic | O18 | 95 | O18 | H7 | G2_KL8A1_K1 | 18 |
| 1262556 | 2 | Hemolytic | O18 | 95 | O18 | H7 | G2_KL8A1_K1 | 18 |
| 1262556 | 2 | Hemolytic | O18 | 95 | O18 | H7 | G2_KL8A1_K1 | 18 |
| 1262556 | 7 | Hemolytic | O18 | 95 | O18 | H7 | G2_KL8A1_K1 | 18 |
| 1262556 | 7 | Hemolytic | O18 | 95 | O18 | H7 | G2_KL8A1_K1 | 18 |
| 1262351 | 2 | Hemolytic | O18 | 95 | O18 | H7 | G2_KL8A1_K1 | 18 |
| 1262351 | 2 | Hemolytic | O18 | 95 | O18 | H7 | G2_KL8A1_K1 | 18 |
| 1262351 | 7 | Hemolytic | O18 | 95 | O18 | H7 | G2_KL8A1_K1 | 18 |
| 1262351 | 7 | Hemolytic | O18 | 95 | O18 | H7 | G2_KL8A1_K1 | 18 |
| 1262491 | 2 | Non-Hemolytic | non-9V | 602 | OgN17 | H21 | group1_CL35_ | 86 |
| 1262491 | 2 | Non-Hemolytic | non-9V | 602 | OgN17 | H21 | group1_CL35_ | 86 |
| 1262491 | 7 | Non-Hemolytic | non-9V | 602 | OgN17 | H21 | group1_CL35_ | 86 |
| 1262491 | 7 | Non-Hemolytic | non-9V | 602 | OgN17 | H21 | group1_CL35_ | 86 |
| 1262696 | 2 | Hemolytic | O18 | 95 | O18 | H7 | G2_KL8A1_K1 | 18 |
| 1262696 | 2 | Hemolytic | O18 | 95 | O18 | H7 | G2_KL8A1_K1 | 18 |
| 1262696 | 7 | Non-Hemolytic | non-9V | 602 | OgN17 | H21 | group1_CL35_ | 86 |
| 1262696 | 7 | Non-Hemolytic | non-9V | 602 | OgN17 | H21 | group1_CL35_ | 86 |
| 1262696 | 14 | Non-Hemolytic | non-9V | 602 | OgN17 | H21 | group1_CL35_ | 86 |
| 1262696 | 14 | Non-Hemolytic | non-9V | 602 | OgN17 | H21 | group1_CL35_ | 86 |
| 1262891 | 2 | Hemolytic | O18 | 95 | O18 | H7 | G2_KL8A1_K1 | 18 |
| 1262891 | 2 | Hemolytic | O18 | 95 | O18 | H7 | G2_KL8A1_K1 | 18 |
| 1262891 | 7 | Hemolytic | O18 | 95 | O18 | H7 | G2_KL8A1_K1 | 18 |
| 1262891 | 7 | Hemolytic | O18 | 95 | O18 | H7 | G2_KL8A1_K1 | 18 |
| 1262769 | 2 | Hemolytic | O18 | 95 | O18 | H7 | G2_KL8A1_K1 | 18 |
| 1262769 | 2 | Hemolytic | O18 | 95 | O18 | H7 | G2_KL8A1_K1 | 18 |
| 1262769 | 7 | Hemolytic | O18 | 95 | O18 | H7 | G2_KL8A1_K1 | 18 |
| 1262769 | 7 | Hemolytic | O18 | 95 | O18 | H7 | G2_KL8A1_K1 | 18 |
| 1262840 | 2 | Hemolytic | O18 | 95 | O18 | H7 | G2_KL8A1_K1 | 18 |
| 1262840 | 2 | Hemolytic | O18 | 95 | O18 | H7 | G2_KL8A1_K1 | 18 |
| 1262840 | 7 | Hemolytic | O18 | 95 | O18 | H7 | G2_KL8A1_K1 | 18 |
| 1262840 | 7 | Hemolytic | O18 | 95 | O18 | H7 | G2_KL8A1_K1 | 18 |
| 1262840 | 14 | Hemolytic | O18 | 95 | O18 | H7 | G2_KL8A1_K1 | 18 |
| 1262840 | 14 | Hemolytic | O18 | 95 | O18 | H7 | G2_KL8A1_K1 | 18 |
